# Examining the neural mechanisms of rTMS: a naturalistic pilot study of acute and serial effects in pharmacoresistant depression

**DOI:** 10.3389/fncir.2023.1161826

**Published:** 2023-05-03

**Authors:** Camila Cosmo, Amin Zandvakili, Nicholas J. Petrosino, Thaise Graziele L. de O. Toutain, José Garcia Vivas Miranda, Noah S. Philip

**Affiliations:** ^1^Department of Psychiatry and Human Behavior, The Warren Alpert Medical School, Brown University, Providence, RI, United States; ^2^VA RR&D Center for Neurorestoration and Neurotechnology, VA Providence Healthcare System, Providence, RI, United States; ^3^Institute of Health Sciences, Federal University of Bahia, Salvador, Brazil; ^4^Institute of Physics, Federal University of Bahia, Salvador, Brazil

**Keywords:** functional cortical networks, repetitive transcranial magnetic stimulation (rTMS), non-invasive brain stimulation (NIBS), pharmacoresistant depression, treatment-resistant depression (TRD), qEEG, power spectral analysis (FFT), naturalistic study

## Abstract

**Introduction:**

Previous studies have demonstrated the effectiveness of therapeutic repetitive transcranial magnetic stimulation (rTMS) to treat pharmacoresistant depression. Nevertheless, these trials have primarily focused on the therapeutic and neurophysiological effects of rTMS following a long-term treatment course. Identifying brain-based biomarkers of early rTMS therapeutic response remains an important unanswered question. In this pilot study, we examined the effects of rTMS on individuals with pharmacoresistant depression using a graph-based method, called Functional Cortical Networks (FCN), and serial electroencephalography (EEG). We hypothesized that changes in brain activity would occur early in treatment course.

**Methods:**

A total of 15 patients with pharmacoresistant depression underwent five rTMS sessions (5Hz over the left dorsolateral prefrontal cortex, 120%MT, up to 4,000 pulses/session). Five participants received additional rTMS treatment, up to 40 sessions. Resting EEG activity was measured at baseline and following every five sessions, using 64-channel EEG, for 10 minutes with eyes closed. An FCN model was constructed using time-varying graphs and motif synchronization. The primary outcome was acute changes in weighted-node degree. Secondary outcomes included serial FFT-based power spectral analysis and changes in depressive symptoms measured by the 9-Item Patient Health Questionnaire (PHQ-9) and the 30-item Inventory of Depressive Symptoms-Self Report (IDS-SR).

**Results:**

We found a significant acute effect over the left posterior area after five sessions, as evidenced by an increase in weighted-node degree of 37,824.59 (95% CI, 468.20 to 75,180.98) and a marginal enhancement in the left frontal region (t (14) = 2.0820, *p* = 0.056). One-way repeated measures ANOVA indicated a significant decrease in absolute beta power over the left prefrontal cortex (F (7, 28) = 2.37, *p* = 0.048) following ten rTMS sessions. Furthermore, a significant clinical improvement was observed following five rTMS sessions on both PHQ-9 (t (14) = 2.7093, *p* = 0.017) and IDS-SR (t (14) = 2.5278, *p* = 0.024) and progressed along the treatment course.

**Discussion:**

Our findings suggest that FCN models and serial EEG may contribute to a deeper understanding of mechanisms underlying rTMS treatment. Additional research is required to investigate the acute and serial effects of rTMS in pharmacoresistant depression and assess whether early EEG changes could serve as predictors of therapeutic rTMS response.

## Introduction

Recent data from the World Health Organization's Global Health Estimate report indicate that depressive disorders are a significant public health concern that affects a substantial proportion of the American population, with a prevalence of 5.9% (World Health Organization, 2017). Specifically, these data suggested that 8.4% of Years Lived with Disability and Disability-Adjusted Life Years could be attributed to the impact of depression, indicating a significant burden on the population (World Health Organization, 2017). Furthermore, the Centers for Disease Control and Prevention's 2019 National Health Interview Survey reported that 4.7% of adults aged 18 or older exhibited regular symptoms of depression, and 18.5% experienced some degree of depressive symptoms (Clarke et al., 2019). The 2020 National Survey on Drug Use and Health also found a high prevalence of Major Depressive Disorder (MDD), with an estimated 8.4% of the adult population affected (Center for Behavioral Health Statistics and RTI International, 2021). In addition, the survey revealed that 6% of U.S. adults experienced at least one episode of depression with severe impairment (Center for Behavioral Health Statistics and RTI International, 2021). Notably, among individuals with a severe presentation, 71% underwent treatment (Center for Behavioral Health Statistics and RTI International, 2021). The absence of a standardized definition for pharmacoresistant depression, also referred to as treatment-resistant depression (TRD), has led to considerable variation in its reported prevalence across the literature (Zhdanava et al., 2021). In a recent study, Zhdanava et al. found that the estimated annual prevalence of American adults undergoing pharmacotherapy for MDD was 8.9 million, with 30.9% of these individuals presenting TRD (Zhdanava et al., 2021). Although there is no consensus regarding the concept of pharmacoresistant depression, it is generally defined as the lack of remission of depressive symptoms following a minimum of two optimal trials of evidence-based pharmacological treatment (McIntyre et al., 2014; Cosmo et al., 2021; Zhdanava et al., 2021; Denee et al., 2022).

Repetitive transcranial magnetic stimulation (rTMS) is a non-invasive technique that induces an electrical current in the cortex through magnetic pulses, modifying brain networks (Carpenter et al., 2012; Gaynes et al., 2014; Cosmo et al., 2021). In 2008, the US Food and Drug Administration cleared rTMS for the treatment of pharmacoresistant depression. A pivotal study, a large multisite trial comprising 307 participants, assessed the effectiveness of therapeutic rTMS in treatment-resistant depression utilizing a naturalistic approach (Carpenter et al., 2012). It consisted of an acute phase treatment with an average of 28.3 rTMS sessions [with a standard deviation (SD) of 10.1]. The primary outcome measure was the Clinical Global Impressions-Severity of Illness scale. A significant improvement in depression symptoms and severity was observed from baseline to endpoint. The findings of this study are consistent with subsequent controlled trials and support the use of rTMS as an effective, safe, and well-tolerated treatment for TRD (Avery and Holtzheimer, 2006; Demitrack and Thase, 2009; Carpenter et al., 2012; Connolly et al., 2012; Benadhira et al., 2017; Cosmo et al., 2021).

Since its initial clearance, rTMS has been increasingly utilized in TRD (Carpenter et al., 2012; Gaynes et al., 2014; Anderson et al., 2016; Cosmo et al., 2021), with emerging data supporting its use in a broad range of neuropsychiatric disorders (Carpenter et al., 2012; Anderson et al., 2016; Zandvakili et al., 2019; Alyagon et al., 2020; Cosmo et al., 2021; McIntyre et al., 2021; Khedr et al., 2022). Despite its clinical successes, the mechanism of action of rTMS and its effects during treatment have yet to be fully elucidated. Prior studies utilizing quantitative electroencephalography (qEEG) yielded promising findings of underlying mechanisms of rTMS (Spronk et al., 2008; Valiulis et al., 2012; Noda et al., 2013; Wozniak-Kwasniewska et al., 2015; Kallioniemi and Daskalakis, 2022; Morris et al., 2023). The effects of high-frequency and low-frequency rTMS protocols on EEG power spectral analysis were assessed in a sample of 45 patients with TRD. While clinical efficacy was similar for both frequencies, distinct electrophysiological measures were observed. The low-frequency group showed increased frontal alpha power asymmetry toward the right hemisphere and higher beta power in frontal, central, parietal, and left temporal areas. In contrast, the high-frequency group demonstrated more widespread changes, including increased delta power in the left hemisphere, and increased alpha power in the right (Valiulis et al., 2012). Similar to other rTMS studies, this trial has primarily applied a pre- vs. post-treatment comparison (Spronk et al., 2008; Valiulis et al., 2012; Noda et al., 2013; Morris et al., 2023). Since clinical improvement does not always progress linearly over time, multiple EEG acquisitions over the course of rTMS may yield novel observations that may enhance our understanding of mechanisms underlying TMS and inform the development of novel treatment protocols. Furthermore, the identification of brain-based biomarkers of early therapeutic response remains an important and unanswered question in the field.

Graph theory provides a robust framework for analyzing brain networks (Sporns, 2018). Using graph-based analytical methods, such as functional cortical network (FCN), it is possible to investigate the connectivity patterns among distinct brain regions, and how these patterns evolve over time in response to interventions or stimuli (Sporns, 2018). Graph-based models have demonstrated promising results as an approach to characterizing brain connectivity patterns in neuropsychiatric disorders (Toutain et al., 2022, 2023), and evaluating acute neurophysiological effects following brain stimulation (Polania et al., 2011; Cosmo et al., 2015).

To this end, we utilized Functional Cortical Networks and EEG power spectral analysis, to examine the acute and serial effects of rTMS on patients diagnosed with treatment-resistant depression. qEEG and FCN are two distinct but complementary approaches for investigating brain dynamics. Combining these methods may lead to a comprehensive understanding of the neural mechanisms underlying rTMS effects. We hypothesized that the therapeutic effects of repetitive transcranial magnetic stimulation on treatment-resistant depression would be observable early in the treatment course, as assessed by FCN, qEEG, and standardized clinical assessment scales.

## Materials and methods

This naturalistic pilot study was conducted at the VA Providence Medical Center upon chart review of patients with pharmacoresistant depression that underwent rTMS treatment from October 2018 to December 2020. Methods were approved by VA Providence Institutional Review Board.

### Participants

Fifteen veterans (12 males and 3 females) with pharmacoresistant depression (mean age ± SD: 52 ± 10.5 years) were enrolled in this study. Inclusion criteria were (1) a primary diagnosis of MDD consistent with the Diagnostic and Statistical Manual of Mental Disorders, fifth edition (DSM-V), and confirmed by an experienced psychiatrist; (2) had failed at least two antidepressant trials; and (3) were recommended by their primary providers to undergo rTMS as a therapeutic strategy. The exclusion criteria were (1) former or current presence of psychotic features or diagnosis of primary psychotic disorder; (2) cognitive impairment; (3) having any contraindication for iTBS (e.g., implanted devices/metal, pregnancy, unstable medical conditions, history of seizure, etc.); or (4) active suicidality. Given the naturalistic nature of this study, participants continued in any ongoing treatment (i.e., medications, therapy, etc.) while having adjunctive rTMS sessions. Consistent with clinical practice, other treatments were largely held stable during rTMS unless clinically indicated.

### Procedures and TMS parameters

In the acute effects group, participants underwent five sessions of rTMS at 5 Hz, 120% of motor threshold (MT), and up to 4,000 pulses per session. The number of pulses varied from 3,000 to 4,000 according to psychiatrist-advised protocol and patients' tolerability. Due to the time required for serial EEG recording, five male participants (mean age ± SD: 59 ± 6.93 years) agreed to receive additional rTMS treatment, with the same parameters, in a five consecutive sessions-block (up to 40 sessions), forming the serial effects subgroup. The remaining ten patients continued the rTMS treatment course without EEG recording.

After MT determination, the coil was placed over the left dorsolateral prefrontal cortex (DLPFC), approximately between F3 and F5 electrode locations (using the international 10/20 EEG system). rTMS was delivered by applying the Magstim^®^ Rapid^2^ Plus^1^ system (Magstim, UK).

The left dorsolateral prefrontal cortex, a known pathophysiological target of depression, has been shown to be deeply connected with limbic structures responsible for regulating mood (Pandya et al., 2012; Zhang et al., 2018). High-frequency TMS delivered at this region has been associated with polysynaptic effects and, consequently, effective reduction of depressive symptoms (Philip et al., 2015; Cosmo et al., 2021).

### Outcome measures

#### EEG acquisition and analysis

Ten minutes eyes closed, resting-state EEG was recorded at baseline, and at the end of each block of five rTMS sessions. The brain electrical activity was recorded by a 64-channel EEG cap, placed in accordance with the 10-10 system, an extension of the international 10–20 system, with CPz as reference, and using an EEG gel system (eego, ANT, Enschede, the Netherlands) with Ag/AgCl electrodes. Electrode-skin impedance was set below 20 kΩ, and data were sampled at 600 Hz.

EEG preprocessing and analysis were performed using EEGLAB, running on MATLAB [R2021b (9.11), The Mathworks, Inc.]. EEG signals were filtered with a band-pass filter ranging from 0.5 to 50 Hz and segmented into 1s epochs. Independent Component Analysis (ICA) was automatically performed to remove ocular, electrocardiographic, and electromyographic artifacts. In addition, an automatic procedure was employed to reject epochs containing signal amplitudes >100 μV or <-100 μV. Subsequently, a manual visual inspection was carried out to eliminate any remaining artifacts. Then, power spectral density (PSD) analyses of artifact-free 1s epochs (without overlap) were carried out by applying customed MATLAB scripts. We applied Welch's power spectral density to estimate the mean PSD for all epochs of the signal. The average PSD values for each frequency band were estimated. Power was calculated for the following four frequency bands: delta (1–4 Hz), theta (4–8 Hz), alpha (8–13 Hz), and beta (13–30 Hz). For Fast Fourier Transform (FFT) analyses, the Bonferroni procedure was applied to correct for multiple comparisons across the four frequency bands.

In summary, EEG data were used to (1) build functional cortical networks to assess the acute effects of rTMS on the connectivity between the brain regions, and (2) compute power spectral changes in a series analysis of rTMS effects at the end of every 5-session block (up to 8 blocks).

#### Functional cortical network model

An FCN model was developed by using time-varying graphs (TVG) and motif synchronization methods (Rosário et al., 2015). It consists of applying local oscillation patterns (motifs) to synchronize traces of pairs of EEG channels over time, creating a TVG. This graph includes a set of nodes and edges—the first corresponding to the electrodes, and the second representing the synchronization between these electrode regions. Each pair of electrodes are compared in a defined time window (1,000 ms), and for every window, a network (i.e., graph) is constructed, and connectivity is estimated for each electrode. Based on a sliding time window along the EEG recording, 72,000 graphs were generated and overlapped, creating a weighted static aggregate network. On this weighted network, weights indicate the number of connections over time; namely, the number of times synchronization between pairs of electrodes occurred. For this study, functional cortical networks were developed based on the following parameters: threshold (0.80), window length (60 points); lag window (1), τ minimum (3), τ maximum (15), TVG step (1), motif lag (1), sample rate (600 Hz), resulting in 72,000 points for a total period of 120,000 ms. These parameters were applied to ensure that the resulting synchronization had only a 1% chance of being due to chance. Two resting state networks, pre- and post- 5 rTMS sessions block, were created for each patient.

FCN analyses were conducted by grouping electrodes into clusters based on corresponding brain regions as follows: left frontal: AF7, AF3, F7, F5, F3, F1, FC5, FC3, FC1 electrodes; right frontal: AF8, AF4, F8, F6, F4, F2, FC6, FC4, FC2; left centrotemporal: T7, C5, C3, C1, TP7, CP5, CP3, CP1; right centrotemporal: T8, C6, C4, C2, TP8, CP6, CP4, CP2; left posterior: P7, P5, P3, P1, PO7, PO5, PO3, O1; and right posterior: P8, P6, P4, P2, PO8, PO6, PO4, O2. Electrode grouping was performed for the computation of topological indices of the functional cortical networks. Specifically, the indices were estimated for each electrode separately and subsequently averaged across electrodes within each region. Therefore, regional connectivity represents the average connectivity of the corresponding electrodes.

Our primary outcome was acute changes in weighted-node degree. This metric reflects how many times and how long the nodes (i.e., EEG electrodes) were synchronized over time, displaying the network evolution after five rTMS sessions, which we refer to as acute changes. For further information on the FCN methods, please refer to (Cosmo et al., 2015; Rosário et al., 2015). Secondary outcomes included serial FFT-based power spectral analysis and changes in depressive symptoms severity as measured by the 9-Item Patient Health Questionnaire (PHQ-9) and the 30-item Inventory of Depressive Symptoms-Self Report (IDS-SR), as further explained below.

#### Self-reported clinical rating scales

Depressive symptom severity was measured by applying the PHQ-9 and IDS-SR (Gili et al., 2011). These clinical scales are sensitive instruments and can be applied in a one-week window to assess changes in symptom severity. They were designed to grade the nine main depressive symptomatology domains in accordance with the Diagnostic and Statistical Manual of Mental Disorders, fourth edition (DSM-IV), with the IDS-SR having additional items to rate melancholic and atypical features. The IDS-SR total score ranges from 0 to 84, with a rating of 18 or more implying clinically significant depression, 39–48 indicating severe presentation, and above 49, very severe symptomatology (Gili et al., 2011). As far as PHQ-9, its total score varies from 0 to 27, with a result between 10 and 14 indicating moderate depression, 15 and 19 implying moderately severe depression, and a score of 20 or more suggesting severe depression.

### Statistical analysis

Clinical and demographic features were assessed using descriptive statistical procedures such as central tendency and dispersion measures. The Shapiro–Wilk test was used to assess the data normality, and Mauchly's sphericity test was applied, determining that the assumption of sphericity was met as required for the repeated-measures ANOVA. Parameters of all the electrodes were analyzed for each individual, and a weighted-node degree was generated for each electrode, with *p*-values corrected using the Bonferroni technique. The primary outcome measure, weighted-node degree, was analyzed by applying paired *t*-test to compare within-group changes (i.e., baseline vs. the fifth rTMS session). One-way repeated measures ANOVA was performed to assess serial clinical and absolute/relative power data. Power was calculated based on FFT analysis, with absolute and relative power estimated for each frequency band. For the FFT analyses, the Bonferroni procedure was applied to correct for multiple comparisons across the four frequency bands. Additional paired *t*-tests were carried out to compare baseline (T0) and post (T1, end of 5 rTMS sessions; T2, after 10 sessions; T3, 15 sessions; T4, 20 sessions; T5, 25 sessions; T6, after last rTMS session; and T7, 1 week after the end of treatment) data of each secondary outcome within-group. Statistical analyses were performed using the Stata software program, version 16.1 (StataCorp LP, College Station, TX, USA). Statistical significance was determined at alpha = 5%, and all p-values were two-tailed.

## Results

### Demographic and clinical characteristics

Baseline demographic features for both groups—(a) rTMS acute effects (*n* = 15); and (b) rTMS serial effects (SE; *n* = 5) are shown in Table 1. The severity of depressive symptoms was similar for both groups at baseline, indicating moderately severe depression, based on PHQ-9 measures (acute effects: 15.4 ± 6.71; serial effects: 16.4 ± 4.83) and severe presentation according to IDS-SR scores (acute effects: 41 ± 13.54; serial effects: 39.2 ± 7.08) (Tables 2, 3).

**Table 1:** Demographic features at baseline.

	**Acute effects (*n =* 15)**	**Serial effects (*n =* 5)**
Age (years)^a^	52 (10.53)	59 (6.93)
Female sex (%)	20	0
**Race (%)**		
African American	0	0
American Indian/Alaska native	0	0
Multiracial	7.69	0
White	92.31	100
**Ethnicity**		
Not of Hispanic origin	93.33	100
Hispanic origin	6.67	0
**Marital status** ^b^		
Single	20	0
Married	33.33	60
Separated	6.67	0
Divorced	33.33	40
**Employment status** ^b^		
Full time	33.33	40
Part time	6.67	0
Unemployed	13.33	0
Multiple status	20	0
Service-connected disability (mental health)	78.57	80

a Age presented as mean ± standard deviation (SD).

b Totals do not sum up to 100% due to participants non-response.

**Table 2:** Acute effects of rTMS on clinical features.

	**T0**	**T1**	**Within-groups (p)^‡^**
PHQ-9^a^	15.4 (6.71)	13.5 (6.16)	0.017
IDS-SR^a^	41 (13.54)	34.4 (12.76)	0.024

a Clinical variables of 15 participants described as mean ± SD; PHQ-9, 9-Item Patient Health Questionnaire; IDS-SR, 30-item Inventory of Depressive Symptoms-Self Report; rTMS, repetitive Transcranial Magnetic Stimulation; SD, Standard deviation.

‡ p-values correspond to paired t-test.

**Table 3:** Serial effects of rTMS on clinical features.

	**T0**	**T1**	**T2**	**T3**	**T4**	**T5**	**T6**	**T7^#^**	**Within-groups (p)^‡^**
PHQ-9^a^	16.4 (4.83)	14.8 (5.40)	10.6 (7.44)	12 (7.31)	10 (9.25)	9.4 (9.50)	9 (8.69)	8.2 (9.18)	0.001
IDS-SR^a^	39.2 (7.08)	35 (16.11)	31.2 (14.10)	30 (13.91)	28.6 (15.34)	26 (16.14)	24.8 (16.81)	20.8 (16.75)	<0.001

a Clinical variables of 5 participants described as mean ± SD; PHQ-9, 9-Item Patient Health Questionnaire (); IDS-SR, 30-item Inventory of Depressive Symptoms-Self Report; rTMS, repetitive Transcranial Magnetic Stimulation; SD, Standard deviation.

# One week after the last rTMS session.

‡ p-values correspond to repeated measures ANOVA.

### Acute effects—FCN

**Figure 1:**
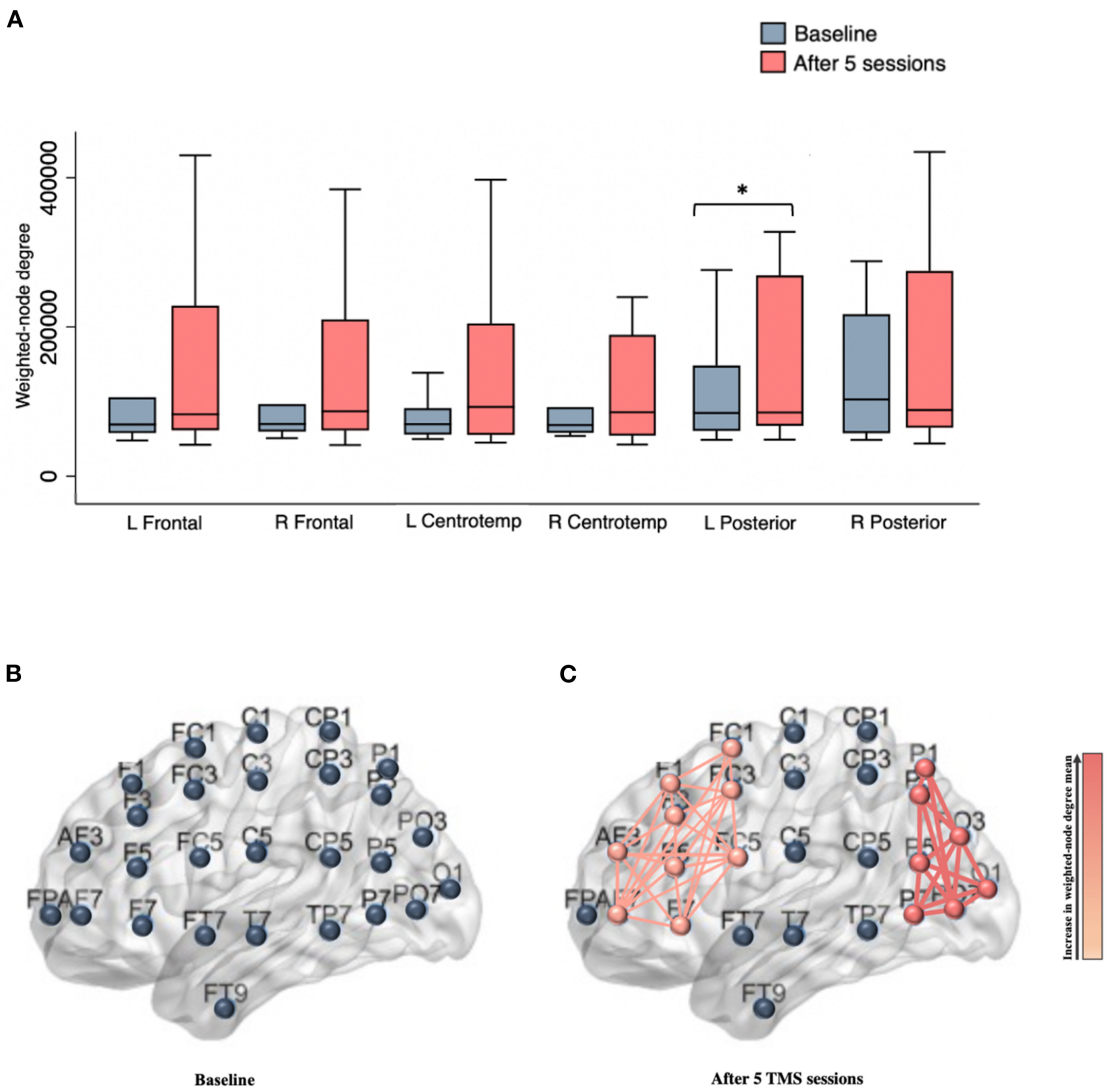
**(A)** Box plots show the weighted-node degree mean for brain regions at baseline and after five rTMS sessions. An acute effect was observed over the left posterior area, evidenced by a statistically significant increase in the weighted-node degree mean (*p* = 0.047); **(B)** Illustration showing left electrodes (e.g., nodes) distribution; **(C)** Schematic representation of left frontal and posterior clusters following five rTMS sessions. The thickness of the edges and the gradient color represents the strength of association between the network nodes. L, left; R, Right; Centrotemp, centrotemporal. ^*^Indicates statistical significance.

After five rTMS sessions, an acute effect was observed over the left posterior area, evidenced by a statistically significant increase of 37,824.59 in the weighted-node degree mean of the electrodes located in this region [95% CI, 468.20 to 75,180.98, t (14) = 2.172, *p* = 0.047, with a medium effect size (*d* = 0.561)]. Increased synchronization was also noted in the left frontal electrodes [t (14) = 2.082, *p* = 0.056; medium effect size (*d* = 0.537)], although with nominal statistical significance (Figure 1).

### Serial effects—Power spectral analysis

**Figure 2:**
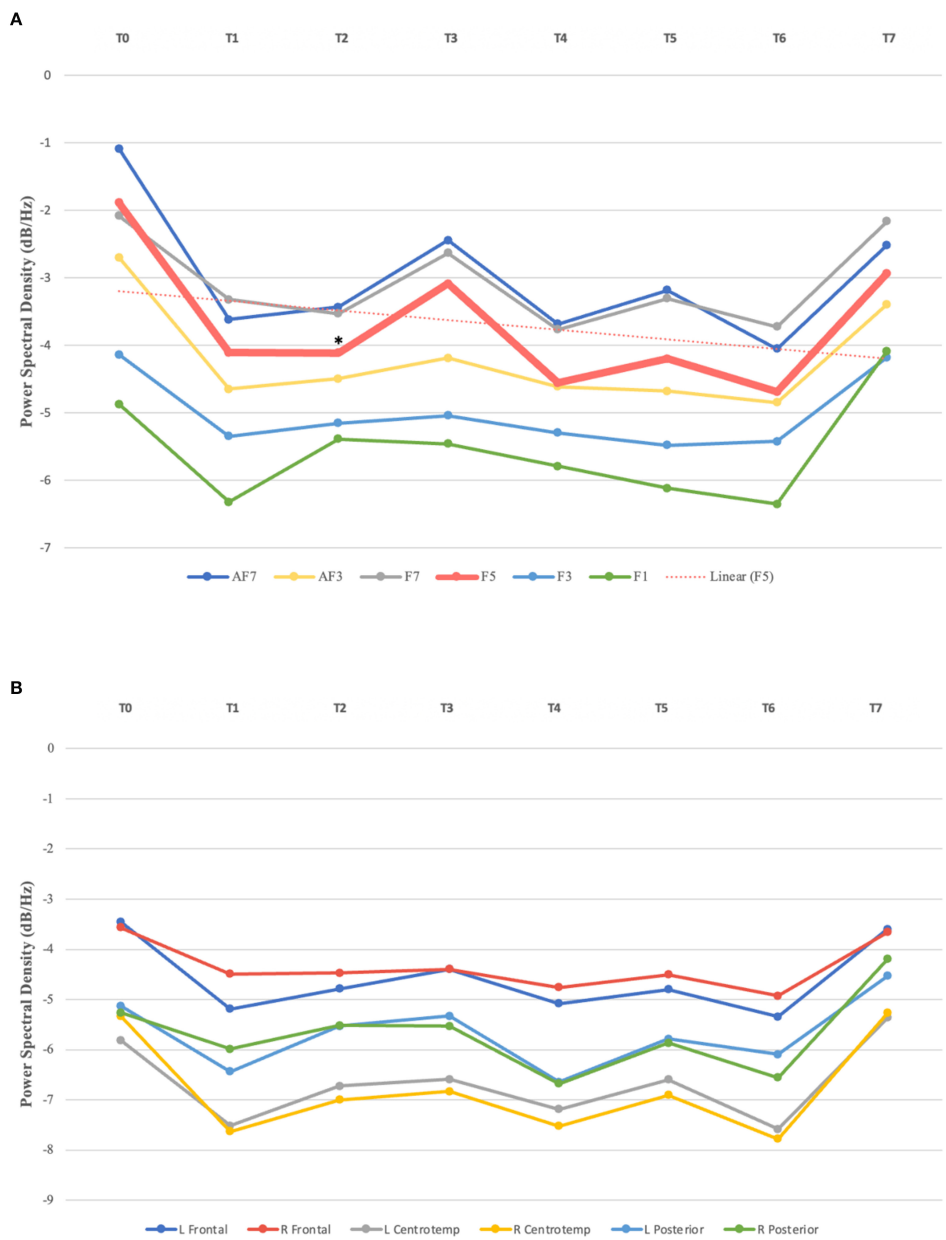
**(A)** Absolute beta power in left prefrontal channels over time. Statistically significant absolute power decrease observed in F5 after 10 sessions (T2), compared to baseline (T0); linear trendline for F5. **(B)** Absolute beta power in brain regions from T0–T7. L, left; R, Right; Centrotemp, centrotemporal. ^*^Indicates statistical significance.

One-way repeated measures ANOVA showed a statistically significant decrease in the absolute power in the beta band over the left prefrontal cortex [F (7, 28) = 2.37, *p* = 0.048], with notable effects observed as early as after 10 rTMS sessions [t (4) = 3.225, *p* = 0.032; with a large effect size (*d* = −1.442)]. Upon analysis of each prefrontal EEG channel, an absolute beta power decrease was mainly observed in F5 [F (7, 28) = 2.85, *p* = 0.022], with a significant reduction at T2 [t (4) = 2.922, *p* = 0.043; with a large effect size (*d* = −1.307)], compared to baseline (T0); and there was no significant difference in the beta power at other brain regions (*p* ≥ 0.05) (Figure 2). In addition, no significant changes were observed in the absolute or relative power in the delta, theta, or alpha bands following the stimulation sessions (*p* ≥ 0.05).

### Clinical effects—PHQ-9 and IDS-SR

To assess whether the acute and serial neurophysiological effects of rTMS treatment reflected changes in depressive symptoms, clinical outcomes (PHQ-9 and IDS-SR) were analyzed in both groups [acute effects (*n* = 15) and serial effects (*n* = 5)].

Consistent with the FCN findings, a statistically significant clinical improvement was observed following five rTMS sessions. The acute effects were observed on both the PHQ-9 [t (14) = 2.709, *p* = 0.017; *d* = 0.699] and IDS-SR [t (14) = 2.528, *p* = 0.024; d = 0.653] (Table 2).

Concerning rTMS serial effects on clinical outcomes, a significant improvement was noted over time on both the PHQ-9 [F (7, 28) = 4.93, *p* = 0.001] and IDS-SR scales [F (7, 28) = 5.82, *p* < 0.001] (Table 3). Interestingly, PHQ-9 was more sensitive in detecting early clinical response, as improvement was observed following 10 sessions, corresponding to the observed absolute beta power reduction at the same timepoint (T2). In contrast, clinical improvement as measured by the IDS-SR was not noted until after 15 sessions. Despite this finding, PHQ-9 only detected statistically significant clinical improvement at specific time points (T2, T6, and T7). IDS-SR demonstrated a greater consistency in detecting the longitudinal effects, with a sustained significant response observed over treatment.

## Discussion

This study indicates that FCN models may provide a sensitive measure of acute changes in neural mechanisms underlying therapeutic rTMS. Five rTMS sessions were sufficient to evoke higher synchronization between electrodes in the left posterior and prefrontal areas, with a statistically significant increase observed in the first. Additionally, this pilot data indicates the potential of serial EEG to monitor rTMS-induced changes in cortical networks during the treatment course. Our results demonstrate that rTMS elicited a reduction in beta power over time in the left prefrontal area, starting as early as after the first ten sessions. In line with the FCN and qEEG findings, statistically significant clinical improvement was observed following five and ten rTMS sessions, respectively. Clinical naturalistic therapeutic response was observed over time on both—the PHQ-9 and IDS-SR, with the latter being more consistent in detecting longitudinal improvement over the course of rTMS treatment. The hypotheses generated by this naturalistic study require further investigation through a more rigorous research design, such as randomized clinical trials, to examine the clinical efficacy of rTMS in individuals with treatment-resistant depression, as well as the corresponding acute and serial neurophysiological changes induced by this neuromodulation technique.

Although rTMS has been widely investigated as a neuromodulatory tool for numerous neuropsychiatric disorders, its neurophysiological mechanisms remain unclear (Carpenter et al., 2012; Anderson et al., 2016; Zandvakili et al., 2019; Alyagon et al., 2020; Cosmo et al., 2021; McIntyre et al., 2021; Khedr et al., 2022). The use of the FCN model in this study is innovative as it offers a novel approach to understanding the acute effects of rTMS on dynamic patterns of brain connectivity over time. This EEG-based model is able to describe brain connectivity by analyzing the temporal synchronization between electrodes (i.e., nodes), providing insight into the evolution of the networks (Cosmo et al., 2015; Rosário et al., 2015). Furthermore, this technique is feasible and cost-effective compared to neuroimaging methods and allows the identification of changes in the cortical connectivity induced by rTMS early on in the course of treatment.

Based on the FCN model, an enhancement in the degree of synchronization in electrodes located in the left posterior and frontal regions was observed, as a result of the administration of five rTMS sessions. Previous studies have demonstrated that rTMS delivered over the left DLPFC led to modulation of brain regions extending beyond the target area, including posterior networks (Liston et al., 2014; Lan et al., 2016; Cardenas et al., 2022), which could be a result of the activation of the central executive network—a frontoparietal system. In our study, a statistically significant increase was noted only in the left posterior area. It is important to note that a statistically significant improvement in clinical outcomes, as quantified by both the PHQ-9 and IDS-SR, was also observed as an acute effect of stimulation. Nevertheless, the potential association between FCN and clinical findings remains uncertain as no correlation analysis was performed given the absence of a linear relationship. Prior trials that have shown that increased left frontal activity might be linked to decreased negative and increased positive affect, while increased left posterior activity is thought to reflect improved emotional processing. Research assessing the relationship between emotional processing and neural networks has established a correlation between the left hemisphere and the experience of positive affect (Spielberg et al., 2008). Previous studies investigating the neural basis of depression have shown a link between depressive symptoms and frontal brain asymmetry, with a reduction in left frontal activity (Henriques and Davidson, 1990, 1991; Eric and Hall, 1999; Palmiero and Piccardi, 2017). It has been suggested that an increase in left frontal activity might be linked to less negative and more positive affect. Hypoactivation of this area has been associated with increased responsivity to negative stimuli, which in turn enhances the likelihood of developing mood disorders, particularly depression (Henriques and Davidson, 1991; Eric and Hall, 1999; Palmiero and Piccardi, 2017). Studies utilizing both EEG and functional magnetic resonance imaging (fMRI) have demonstrated a correlation between left frontal activation and a reduction in negative affect, and an increase in positive affect (Eric and Hall, 1999; Davidson, 2004a,b; Cerqueira et al., 2008; Machado and Cantilino, 2017; Palmiero and Piccardi, 2017). Specifically, fMRI trials have suggested that this link may be mediated by top-down regulation via an inhibitory input to the amygdala (Ochsner et al., 2002; Roalf et al., 2011). Concerning the left posterior brain region, previous research has demonstrated functional impairment in this area in individuals with depression, particularly in the inferior parietal cortex (IPC) (Muller et al., 2013; Mel'nikov et al., 2018). This region plays a crucial role in emotional processing, as well as social cognition, specifically in the affective component of social cognition (Muller et al., 2013; Bzdok et al., 2016; Numssen et al., 2021). It is thought that the dysregulation of the IPC and its resulting impairments in emotional processing and social cognition may be caused by a dysfunction in the connections between the inferior parietal cortex and correlated cortical and subcortical areas (Muller et al., 2013; Numssen et al., 2021).

Although the available data support our findings, it is essential to further investigate the acute effects of rTMS over the left DLPFC and the potential correlation between clinical improvement and the neurophysiological findings observed in our study. The enhancement of synchronization in the left DLPFC, in conjunction with a significant increase in connectivity in the left posterior region, may have led to clinical improvement through different mechanisms. These mechanisms may include an enhancement of positive affect, an improvement in emotional processing and social cognition, or a combination of both. Additional studies are required to determine the existence of a potential clinical-neurophysiological correlation and the precise nature of this relationship.

Furthermore, our results provide evidence for the potential use of serial EEG in monitoring rTMS-induced neurophysiological changes along the treatment course. A reduction in absolute beta power over the left prefrontal cortex was observed as early as after ten sessions, which was consistent with the clinical response as measured by PHQ-9. These findings align with previous research linking beta power to therapeutic response evidenced by reduced depressive symptoms (Paquette et al., 2009). In addition, as suggested by Wyczesany et al., beta frequency band has been associated with negative emotions and increased psychological distress, potentially reflecting automatic responses to negative stimuli (Wyczesany et al., 2018). A recent review of EEG frequency bands in mental health disorders, which analyzed data from 18 studies on depression, concluded that increased absolute beta and theta power, for both eyes-open and eyes-closed conditions, were the main findings in this population (Newson et al., 2018). Taken together, these data support our findings suggesting that the reduction of absolute beta power over the left prefrontal cortex may be associated with improved clinical outcomes in individuals with depression undergoing rTMS treatment.

The current study had several limitations, most prominently (1) the naturalistic design that may have introduced potential sources of observer bias and prevented the use of standardized procedures. Nevertheless, it is noteworthy that the naturalistic data has the advantage of emulating real-life circumstances, providing a high ecological validity, and yielding data that are more generalizable, increasing external validity. By using a naturalistic approach, we aimed to enhance our understanding of how rTMS works in real-world settings, with study participants more accurately representing our clinical population; (2) the small sample size that might have resulted in type II error due to its limited power, also precluding hypothesis testing and more complex analyses; (3) the convenience sample that made the study more prone to sampling bias, and might have introduced confounding factors related to comorbidities and ongoing treatment; (4) population primarily composed of male veterans, possibly affecting the external validity; (5) challenges related to power spectral analysis, particularly its modest spatial resolution and the overlap of spectral properties across some psychiatric disorders; and (6) lack of correction for multiple comparisons across time points and for clinical outcomes, requiring careful interpretation of our findings. As indicated above, veterans received rTMS as an adjunct treatment to ongoing pharmacotherapy, although other treatments remained stable. Furthermore, another important limitation of our study is the lack of a sham control group, which is particularly relevant given the use of self-reported clinical instruments. The potential influence of a placebo effect on these clinical scales needs to be taken into consideration when interpreting our results. Despite these limitations, our pilot study provides an exploratory examination of the effects of rTMS in individuals with pharmacoresistant depression under real-life clinical conditions, along the treatment course.

In summary, this naturalistic pilot study assessed the acute and serial effects of rTMS on brain activity utilizing a Functional Cortical Network model and EEG power spectral analysis, respectively. Our results indicate that FCN models might work as a sensitive measure of acute changes in neural mechanisms underlying therapeutic rTMS. Five rTMS sessions were sufficient to evoke higher synchronization between electrodes in the left posterior and frontal regions, with statistically significant findings observed in the first area. These findings are consistent with previous studies that have demonstrated that increased left posterior activity may reflect improved emotional processing, while enhanced activity in the left frontal region may be associated with improved affect. Furthermore, our results suggest the potential of serial EEG in monitoring rTMS-induced cortical changes throughout the treatment course. Specifically, we observed a reduction in beta power over the left prefrontal cortex as early as after ten sessions, which was consistent with the observed clinical response. This is in line with prior research associating beta power with therapeutic response and support the development of serial EEG as a biomarker of rTMS response, which may aid in tracking and potentially predicting stimulation effects over the course of treatment. These findings may serve as a foundation for future, more rigorous studies utilizing a randomized, double-blind, sham-controlled design to further investigate the acute and serial effects of rTMS in individuals with pharmacoresistant depression and inform the optimization of therapeutic stimulation protocols.

## Data availability statement

Data is available upon request, following to US Department of Veterans Affairs regulations.

## Ethics statement

The studies involving human participants were reviewed and approved by VA Providence Institutional Review Board. The patients/participants provided their written informed consent to participate in this study.

## Author contributions

AZ and NSP designed and conducted the study. CC performed the data analysis and prepared the manuscript draft with intellectual input from AZ, NJP, TT, JM, and NSP. CC, AZ, TT, JM, NJP, and NSP performed a critical review of the manuscript. All authors approved the final manuscript.
